# Human Cytomegalovirus Delays Neutrophil Apoptosis and Stimulates the Release of a Prosurvival Secretome

**DOI:** 10.3389/fimmu.2017.01185

**Published:** 2017-09-25

**Authors:** Joanna M. Pocock, Daniel M. L. Storisteanu, Matthew B. Reeves, Jatinder K. Juss, Mark R. Wills, Andrew S. Cowburn, Edwin R. Chilvers

**Affiliations:** ^1^Department of Medicine, University of Cambridge School of Clinical Medicine, Addenbrooke’s and Papworth Hospitals, Cambridge, United Kingdom; ^2^Department of Virology, Institute of Immunity and Transplantation, University College London, London, United Kingdom; ^3^Department of Physiology, Development and Neuroscience, University of Cambridge, Cambridge, United Kingdom

**Keywords:** human cytomegalovirus, neutrophil, monocyte, polymorphonuclear leukocyte, apoptosis

## Abstract

Human cytomegalovirus (HCMV) is a major cause of viral disease in the young and the immune-suppressed. At sites of infection, HCMV recruits the neutrophil, a cell with a key role in orchestrating the initial immune response. Herein, we report a profound survival response in human neutrophils exposed to the clinical HCMV isolate Merlin, but not evident with the attenuated strain AD169, through suppression of apoptosis. The initial survival event, which is independent of viral gene expression and involves activation of the ERK/MAPK and NF-κB pathways, is augmented by HCMV-stimulated release of a secretory cytokine profile that further prolongs neutrophil lifespan. As aberrant neutrophil survival contributes to tissue damage, we predict that this may be relevant to the immune pathology of HCMV, and the presence of this effect in clinical HCMV strains and its absence in attenuated strains implies a beneficial effect to the virus in pathogenesis and/or dissemination. In addition, we show that HCMV-exposed neutrophils release factors that enhance monocyte recruitment and drive monocyte differentiation to a HCMV-permissive phenotype in an IL-6-dependent manner, thus providing an ideal vehicle for viral dissemination. This study increases understanding of HCMV–neutrophil interactions, highlighting the potential role of neutrophil recruitment as a virulence mechanism to promote HCMV pathology in the host and influence the dissemination of HCMV infection. Targeting these mechanisms may lead to new antiviral strategies aimed at limiting host damage and inhibiting viral spread.

## Introduction

Neutrophils are the most abundant circulating leukocytes and represent the first line of innate immunity against infection. They are terminally differentiated polymorphonuclear granulocytes with a short half-life in the circulation and a propensity to undergoing rapid constitutive apoptosis when aged *in vitro*. This latter response, which is observed during inflammatory resolution *in vivo*, triggers a highly efficient process of macrophage-mediated engulfment or efferocytosis, which prevents these cells from releasing their cytotoxic content (1). Apoptosis is a highly adaptable cellular process influenced by a number of external factors including adhesion (2), hypoxia (3), inflammatory cytokines (4), and microbial agents (5), which mostly act to delay apoptosis and prolong neutrophil survival. While the prolongation of neutrophil lifespan may be critical for efficient handling of bacteria and certain fungi (6), inappropriate neutrophil longevity at sites of inflammation is thought to be pathogenic and contribute to inflammation-mediated damage (7). Conversely, an enhanced susceptibility to apoptosis may contribute to the likelihood of recurrent infections (8, 9), implying that neutrophil apoptosis needs to be tightly regulated to ensure efficient pathogen clearance yet timely and safe immune cell clearance.

Certain bacteria have developed mechanisms to manipulate the apoptotic machinery of the neutrophil, either delaying apoptosis to allow time for intracellular replication (10, 11) and/or using the cell as a “Trojan horse” to facilitate dissemination through the transfer of viable microorganisms within apoptotic neutrophils to efferocytosing macrophages, thus promoting subsequent spread (12, 13).

Several lines of evidence suggest an equally important role for neutrophils during viral infection. Hence, enhanced defensin expression has been shown to aid control of viruses such as HIV, adenovirus, and herpes simplex virus (14–16). Furthermore, recent evidence suggests that neutrophils play a key role in controlling murine CMV replication at secondary sites of infection (17). Neutrophils also attract and stimulate other immune cells both through direct interaction with cells such as dendritic cells (DCs) (18) and through the release of pro-inflammatory chemokines and cytokines (19); this illustrates their potential to orchestrate an adaptive immune response to viral infection.

Human cytomegalovirus (HCMV) is an opportunistic pathogen, which causes severe morbidity in neonates and the immune compromised (20–22). Disease can result from primary infection or reactivation from previously established latent infection and, due to the extensive cellular tropism of the virus, can manifest in a diverse range of tissues (23). Understanding the onset and progression of HCMV pathogenesis is an important area of ongoing research. However, the precise nature of any interaction between HCMV and neutrophils still remains unclear. Neutrophils from healthy latent seropositive patients are HCMV genome negative (24), and although the HCMV antigen pp65 has been detected in the neutrophils of viremic patients (25, 26), this does not reflect a full viral replicative life cycle, as demonstrated by the lack of detectable immediate early (IE) or late viral mRNA (27). Due to the highly phagocytic nature of neutrophils, it has been suggested that intracellular pp65 may in fact represent ingestion of viral material rather than primary lytic infection (27), and the presence of viral DNA in the neutrophil cytoplasm might also be consistent with its presence in phagosomes (28).

Although certain HCMV nucleic acids have been detected in the neutrophils of viremic patients (29, 30), neutrophils appear unable to produce viral transcripts following direct infection *in vitro*, and experimental studies have relied on co-culture of neutrophils with infected endothelial cells (31, 32) or human embryonic lung fibroblasts (33) before any evidence of an abortive infection in neutrophils (31). The short lifespan of neutrophils would also argue against them being a major site of productive infection especially given the protracted life cycle of HCMV; despite this, HCMV carriage in neutrophils can occur in immune-compromised patients and may play a role in a number of CMV disease states including retinitis in late-stage AIDS patients (34, 35). HCMV has also been reported to encode the chemokine mimic viral CXC-chemokine-1 (vCXC-1), a potent chemoattractant for neutrophils (36, 37), and it has been hypothesized that this may support rapid viral dissemination from the site of infection—a characteristic of HCMV.

In this study, we have further investigated the interaction of HCMV with neutrophils. We show that, independently of a co-culture system, cell-free HCMV has a profound effect on neutrophil survival and cytokine expression, independent of viral gene expression but dependent on intracellular signaling involving ERK1/2 and NF-κB, and MCL-1 stabilization. This effect was observed to be viral strain dependent, with the AD169 HCMV strain, which displays a large deletion in the ULb′ genome region, unable to promote neutrophil survival.

Furthermore, the HCMV-stimulated neutrophil secretome was found to have significant paracrine effects on both neutrophils and monocytes. The ensuing neutrophil inflammatory cytokine profile further promotes neutrophil survival, enhances the chemotactic response of autologous monocytes, and promotes monocyte differentiation to a state permissive for HCMV infection. These findings support the hypothesis that the neutrophil–HCMV interaction aids efficient dissemination of the virus, by generating a favorable environment to modulate the phenotype of infiltrating monocytes—the primary vehicle for viral dissemination and persistence in the infected host (38).

## Materials and Methods

### Ethics Statement

The Cambridge Local Research Ethics Committee approved all experiments utilizing primary human cells. The collection of venous blood samples from anonymous donors was performed in accordance with established guidelines for the handling and processing of tissue as defined by the Cambridge Local Research Ethics Committee, including informed written consent (06/Q0108/281).

### Virus Culture and Reagents

The viral isolates TB40/e, Merlin, and AD169 were purified from the supernatants of infected human foreskin fibroblasts (HFFs) using sorbitol gradients as previously described (39) and stored in Iscove’s Modified Dulbecco’s Medium (IMDM) at −70°C. Briefly, viral strains were seeded onto HFF monolayers and cultured in DMEM containing 10% fetal calf serum and 1% penicillin/streptomycin for 10–14 days until 100% cytopathic effect was observed. HFF supernatants containing virus were collected every 48 h and stored at −70°C. Virions were pelleted by centrifugation of supernatants (12,000 rpm, 2 h, 4°C) and then loaded onto six sorbitol gradients (20–70%) before further density centrifugation (20,000 rpm, 1 h, 4°C). Purified virus particles were collected from the 50 and 60% gradients, washed in PBS, and resuspended in IMDM for storage at −70°C as viral inoculum. The viral inoculum was titred by sowing onto a known quantity of HFFs and staining for IE antigen after 24 h to calculate the concentration in plaque-forming units per milliliter, and the volume of viral inoculum calculated to give a standardized MOI was added to neutrophil wells for experiments.

AD169 was obtained from the ATCC and TB40/e from Professor Christian Sinzger (University of Ulm, Germany). WT and mutant Merlin strains were obtained from Dr. Peter Tomasec (University of Cardiff, UK). The Merlin strain deletion mutants were generated by recombineering of the bacterial artificial chromosome of the original Merlin strain (GenBank GU179001.1) as previously described (40), and the genomes were confirmed by Illumina sequencing.

For inactivation of the virus, purified viral stocks were UV irradiated in a class II MSC for 40 min, with the activation status confirmed by inoculation of fresh HFFs and examination for cytopathic effects at 7–14 days postinfection.

### Leukocyte Purification

Primary human blood neutrophils were obtained from healthy adult donors or CMV pneumonitis patients and purified by dextran sedimentation and discontinuous plasma-Percoll™ gradients as previously detailed (41). For protocols requiring ultrapure neutrophils, cells were isolated by semiautomated negative selection using a RoboSep^®^ (StemCell Technologies, Vancouver, BC, Canada), according to the manufacturer’s instructions, with neutrophil purity >98% as determined by light microscopy of cytospins.

CD14+ isolation of human monocytes was performed using a CD14+ cell direct isolation kit (Miltenyi Biotec, Auburn, CA, USA) and magnet-activated cell sorting. Primary CD14+ cells were cultured in X-vivo-15 media (Cambrex, Walkersville, MD, USA) and differentiated into DCs by the addition of interleukin-4 (100 ng/ml) and GM-CSF (100 ng/ml) (Peprotech, Rocky Hill, NJ, USA).

### Leukocyte Culture and Infection

Purified neutrophils were suspended at a final cell concentration of 5 × 10^6^/ml in IMDM (Thermo Fisher Scientific, Loughborough, UK) supplemented with 10% autologous serum and 50 U/ml streptomycin and penicillin (Thermo Fisher Scientific, Loughborough, UK). Cells were plated using cell-saver pipette tips into round-bottom ultra-low attachment 96-well Flexiwell plates (Becton Dickinson, Oxford, UK) and purified cell-free viral inoculum, at a volume calculated to give a standardized MOI, was added to the wells. For experiments involving virion-free inoculum, the inoculum was ultracentrifuged (130,000 *g*, 2 h, 4°C) in a Beckman Coulter Optima ultracentrifuge (Beckman Coulter, Buckinghamshire, UK), to pellet and remove all infectious virions prior to adding to wells. For experiments involving transferrable survival or supernatant effects, purified neutrophils from a second healthy donor, or autologous monocytes, were resuspended in the warmed supernatant harvested from infected neutrophils at 18 h, at a concentration of 5 × 10^6^ cells/ml before plating as described.

For monocyte infections, 5 × 10^6^ monocytes were incubated for 30 min with HCMV and cultured overnight in fresh X-vivo-15 supplemented with 2 mM l-glutamine. For experiments involving inhibitors, compounds or diluent control media were added to wells prior to HCMV addition and were present for the duration of culture.

### Inhibitors, Agonists, and Antibodies

The following agonists were used: Pam3CSK4 (TLR1/2, 0.01–1000 ng/ml, Invivogen, San Diego, CA, USA), ultrapure LPS-EB (TLR4, 10–1000 ng/ml Invivogen, San Diego, CA, USA). The following inhibitors were used: U0126 (ERK1/2, 1 μM, 60 min pre-incubation), PD98059 (MEK1/2, 50 μM), LY294002 (PI3K, 10 μM, 60 min pre-incubation), wortmannin (PI3K, 100 nM), BAY-11-7085 (NF-κB, 3 μM, 60 min pre-incubation), IKK inhibitor VII (IKK, 10 μM), SB203580 (p38, 10 μM), SP600125 (JNK, 5 μM), cytochalasin D (endocytosis, 10 μg/ml; Sigma, Poole, UK), latrunculin A (endocytosis, 10 μM; Sigma, Poole, UK), PAb hTLR2 (TLR2, 10 μg/ml; Invivogen, San Diego, CA, USA), CLI-095 (TLR4, 1 μg/ml, 60 min pre-incubation; Invivogen, San Diego, CA, USA), 324841 (FGF-R1/PDGF-R1/EGF-R, 1 μM, 60 min pre-incubation), actinonin (CD13, 100 nM; Sigma, Poole, UK), and anti-CD13 clone WM15 (CD13, 8 μg/ml) (all Merck Millipore, Nottingham, UK, unless stated). Inhibitors were added 30 min prior to infection unless stated. Wortmannin (100 nM) was added again at 4, 8, and 12 h incubation due to its instability in aqueous solution.

### Immunofluorescence

For immunofluorescent detection of viral IE gene expression, cells were fixed in 70% ethanol (30 min, −20°C) and then incubated with an anti-IE antibody (1:1,000; Millipore, Darmstadt, Germany) followed by Alexafluor 594 nm-conjugated goat anti-mouse antibody (1:1,000; Millipore, Darmstadt, Germany), both for 1 h at room temperature (RT). Nuclei were counterstained with Hoechst (1:1,000; Sigma, Poole, UK).

### Western Blotting

Neutrophils were harvested and analyzed by Western blot using polyclonal antibodies to AKT, phospho-AKT, phospho-ERK1/2, IκB, Mcl-1, Bax, and β-actin (New England Biolabs, Hertfordshire, UK). Detection was performed with the appropriate peroxidase-conjugated secondary antibodies (1:2,000) for 1 h. Chemiluminescence (ECL-plus-kit GE Life Sciences, Buckinghamshire, UK) and exposure to X-ray film (XOMAT-AR; GE life sciences, Buckinghamshire, UK) were used to detect banding.

### Nucleic Acid Isolation, Reverse Transcription, Amplification, and Gel Electrophoresis

RNA (cDNA) and DNA were amplified using the gene-specific primers GAPDH: 5′-GAG TCA ACG GAT TTG GTC GT and 5′-TTG ATT TTG GAGGGA TTC TCG, IE: 5′-CAT CCA CAT CTC CCG CTT AT and 5′-CAC GAC GTT CCT GCA GAC TAT G, in PCR 2x Mastermix (Promega, Madison, WI, USA). The following cycling conditions were applied: 95°C (5 min), then 20–40 cycles of 94°C (40 s), 55°C (40 s), and 72°C (60 s) and a final extension at 72°C for 10 min.

### qPCR Analysis

Total RNA was isolated from neutrophils using TRI-reagent (Sigma, Poole, UK) followed by clean-up and DNase digest using RNeasy column kits (Qiagen). First-strand synthesis was performed with 1 μg of RNA (high-capacity cDNA kit, Applied Biosystems). Relative gene expression was determined by qPCR (ABI) and amplified in Sybr-green master mix (Applied Biosystems) with relevant primers from Qiagen. Gene expression levels were relative to β2 microglobulin using the 2^−CT^ method (42).

### Detection of Neutrophil Apoptosis by Morphology, Electron Microscopy (EM), Flow Cytometry, and Caspase-3 Activity

Neutrophils were harvested at the time points indicated, cytocentrifuged (300 rpm, 3 min, RT) in a Shandon cytospin 3 (Shandon, UK), fixed in methanol, stained with May-Grünwald-Giemsa (Merck Millipore, Nottingham, UK), and examined under oil immersion (×100 objective) light microscopy, with the observer blinded to the experimental condition. Apoptotic neutrophils were defined as those with darkly stained pyknotic nuclei (300 cells counted/slide, 3 slides per condition and time point) (43).

For EM analysis (CM100 transmission electron microscope; Philips, UK), infected neutrophils were harvested by centrifugation, fixed in glutaraldehyde and osmium tetroxide, dehydrated in ethanol, and embedded in Spurr’s resin for sectioning.

Flow cytometric analysis was performed by incubating cells with 5 μl annexin V-FITC and 5 μl propidium iodide for 20 min at 4°C (both BD Biosciences, Oxfordshire, UK). Cells were analyzed by flow cytometry on a BD Accuri™ cytometer (BD Biosciences, Oxfordshire, UK) with 10,000 cells analyzed per condition and data were handled using FlowJo V8.7.1 software (Tree Star, Ashland, OR, USA).

Caspase-3 activity was ascertained on neutrophils incubated with and without HCMV for 18 h by colorimetric caspase-3 assay (Abcam, Cambridgeshire, UK). Neutrophils were cultured for the time period indicated, pelleted, washed in PBS, and resuspended in lysis buffer according to the manufacturer’s instruction, for plate reading to detect cleaved colorimetric caspase-3-substrate at 405 nm.

### Secretome Analysis

Mock and HCMV condition neutrophil supernatants were harvested following 20 h culture and stored at −80°C until use. Some were ultracentrifuged (130,000 *g*, 2 h, 4°C) in a Beckman Coulter Optima ultracentrifuge (Beckman Coulter, Buckinghamshire, UK) prior to storage, to pellet and remove all remaining infectious virions. Depletion of virions from ultracentrifuged supernatants was confirmed by sowing the supernatants onto confluent HFFs and staining for IE antigen after 24 h. The presence of inflammatory mediators in the supernatant of mock and HCMV-treated neutrophils was determined by multispot array using the MesoScale Discovery Platform (Meso Scale Diagnostics, Rockville, MA, USA), with resultant blots quantified by electrochemical luminescence detection.

To assess IL-6 and IL-8 in supernatants, in-house ELISAs were used (44). Briefly, a 96-well microplate was incubated with mouse monoclonal anti-human IL-6 or anti-human IL-8 antibody (2 μg/ml) for 2 h at RT. After washing, wells were blocked using 5% fetal calf serum in PBS-T for 1 h at RT, washed again, and incubated overnight (4°C) with samples and rhIL-6 or rhIL-8 standards. Detection of IL-6 and IL-8 was performed using a biotinylated anti-human IL-6 or anti-human IL-8 detection antibody and ExtrAvidin-AP (Sigma, Poole, UK), followed by p-nitrophenyl phosphate (1 mg/ml in diethanolamine). Plates were read using a Biorad Micro 680 microplate reader at 450 nm.

IL-8 and IL-6 neutralization in neutrophil supernatants was performed using human-IL-8 mAb (1 μg/ml; R&D systems, UK) and human-IL-6 mAb (1 μg/ml; R&D Systems, UK). Antibodies were added to supernatants 60 min prior to the addition of washed protein A micro-beads (Sigma, Poole, UK) for a further 120 min (4°C). The beads were removed by placing vessels in a magnet and IL-6 and IL-8 depletion confirmed by ELISA.

### Cell Migration and Phenotyping Assays

To assess cell migration to supernatants, transwell plates (5 μm pores, 30 μl well volume) (Neuroprobe, USA) were used. Monocytes were labeled using Calcein (BD Biosciences, Oxfordshire, UK). 2 × 10^4^ labeled cells in 20 μl of RPMI 1640 per well were transferred to the transwell plate and incubated at 37°C for 2 h with supernatants from mock or HCMV-infected neutrophils. Migrated cells were counted using an inverted UV microscope (sum of five fields of view from each well, assays performed in triplicate for each donor).

Monocyte immunophenotyping analysis was performed on purified CD14+ cells after 3 days culture in X-vivo-15 media, neutrophil conditioned media, or Merlin-infected neutrophil conditioned media, using antibodies directed against HLA-ABC (MHC class I) and HLA-DR (MHC class II) with the appropriate fluorochrome-conjugated isotype controls (BD Life Sciences, Frankin Lakes, NJ, USA). 10^5^ cells were pelleted (400 *g* for 5 min), resuspended and incubated with the target antibody or control for 20 min, washed once, and resuspended in 500 μl PBS before analyzing by flow cytometry (BD FACScalibur). Data were handled using WinMDI2.9 software.

A mixed leukocyte reaction was performed using 5 × 10^4^ CD14+ monocytes and incubated for 3 days with X-vivo-15 media or supernatants from mock- or HCMV-infected neutrophils. Media was then replaced with fresh media (RPMI-10) supplemented with IL-2, containing 10^5^ purified allogeneic T cells purified from peripheral blood mononuclear cells by negative selection using the RosetteSep Procedure (StemCell Technologies, Grenoble, France). T cell proliferation was quantified by cell counting after 6 days of co-culture.

### Statistical Analysis

Data were plotted as mean ± SEM and analyzed using Prism V6 (GraphPad) software. A paired or unpaired *t*-test was used to evaluate differences in experimental results, or a one-way analysis of variance with *post hoc* significance testing by Dunnet’s test in experiments with >2 variables. Statistical significance was defined as *p* < 0.05.

## Results

### HCMV Promotes Neutrophil Survival Independently of Viral Gene Expression

**Figure 1:**
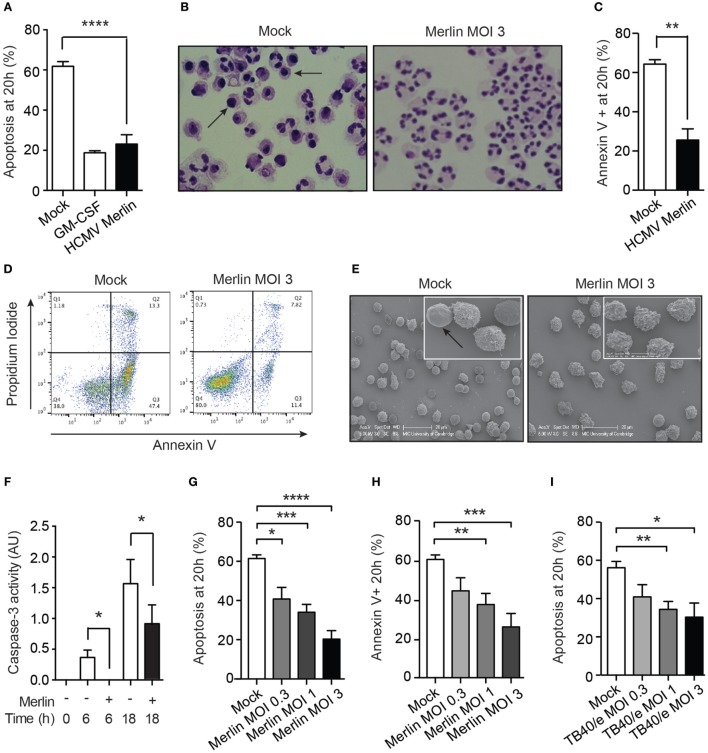
Human cytomegalovirus (HCMV) inhibits the rate of constitutive neutrophil apoptosis. **(A)** Morphological analysis of neutrophil apoptosis in uninfected control (Mock) and infected (HCMV strain Merlin) cells at 20 h postinfection, shown with GM-CSF-treated positive control (MOI 3, *n* = 9), with **(B)** representative photomicrographs of cells with apoptotic cells indicated by arrows (magnification ×1,000). **(C)** Flow cytometry analysis of neutrophil apoptosis in cells infected with Merlin, by annexin V binding at 20 h (MOI 3, *n* = 7), with **(D)** representative flow cytometry plots shown. **(E)** Scanning electron microscopy (EM) images of neutrophils infected with Merlin for 20 h versus control, with smooth apoptotic cells indicated by arrow (bar represents 20 μm). **(F)** Colorimetric assay for caspase-3-activity performed at 0, 6, and 18 h on Mock (−) or HCMV strain Merlin (+) infected cells (MOI 3, *n* = 3). **(G)** Neutrophils infected with Merlin at an MOI of 3, 1, or 0.3 were assayed for apoptosis by morphology (*n* = 5–6) and by **(H)** flow cytometry (*n* = 3–5) at 20 h. **(I)** Neutrophils infected with HCMV strain TB40/e at an MOI of 3, 1, or 0.3 were assayed for apoptosis by morphology at 20 h (*n* = 3–8). *p < 0.05, **p < 0.01, ***p < 0.001, ****p < 0.0001.

Neutrophil co-culture with HCMV-infected fibroblasts has previously been shown to upregulate neutrophil effector function and survival (32). Since clinical strains of HCMV encode chemokines, which promote neutrophil recruitment (17, 45) but only exhibit limited replication in neutrophils, we investigated the nature of the neutrophil interaction with cell-free HCMV, specifically to identify any potential benefit to the virus. We used the Merlin strain that has become the genetic reference for clinical isolates of HCMV (40). Exposure of neutrophils to Merlin protected them from undergoing constitutive time-dependent apoptosis, as judged by morphological analysis under light microscopy (Figure 1A), which revealed a reduction in apoptotic cells containing darkly stained pyknotic nuclei at 20 h (Figure 1B). This was supported by flow cytometry using the apoptosis marker annexin V, which binds to externalized phosphatidylserine on the membrane of apoptotic cells. Co-staining with propidium iodide excluded necrotic cells (Figures 1C,D). EM visually separated the ruffled plasma membrane of viable HCMV-treated cells from the smooth featureless surface of apoptotic cells (Figure 1E). Classically, neutrophil apoptosis requires activation of the caspase cascade, and we show here that HCMV reduced caspase-3 activity at 6 and 18 h postinfection (Figure 1F). Moreover, inhibition of apoptosis occurred in a viral dose-dependent manner, as assessed by both morphology and flow cytometry (Figures 1G,H). Testing of a further HCMV strain, TB40/e, an endothelial-tropic strain of HCMV that infects most cell types (46), revealed an identical dose-dependent effect (Figure 1I).

**Figure 2:**
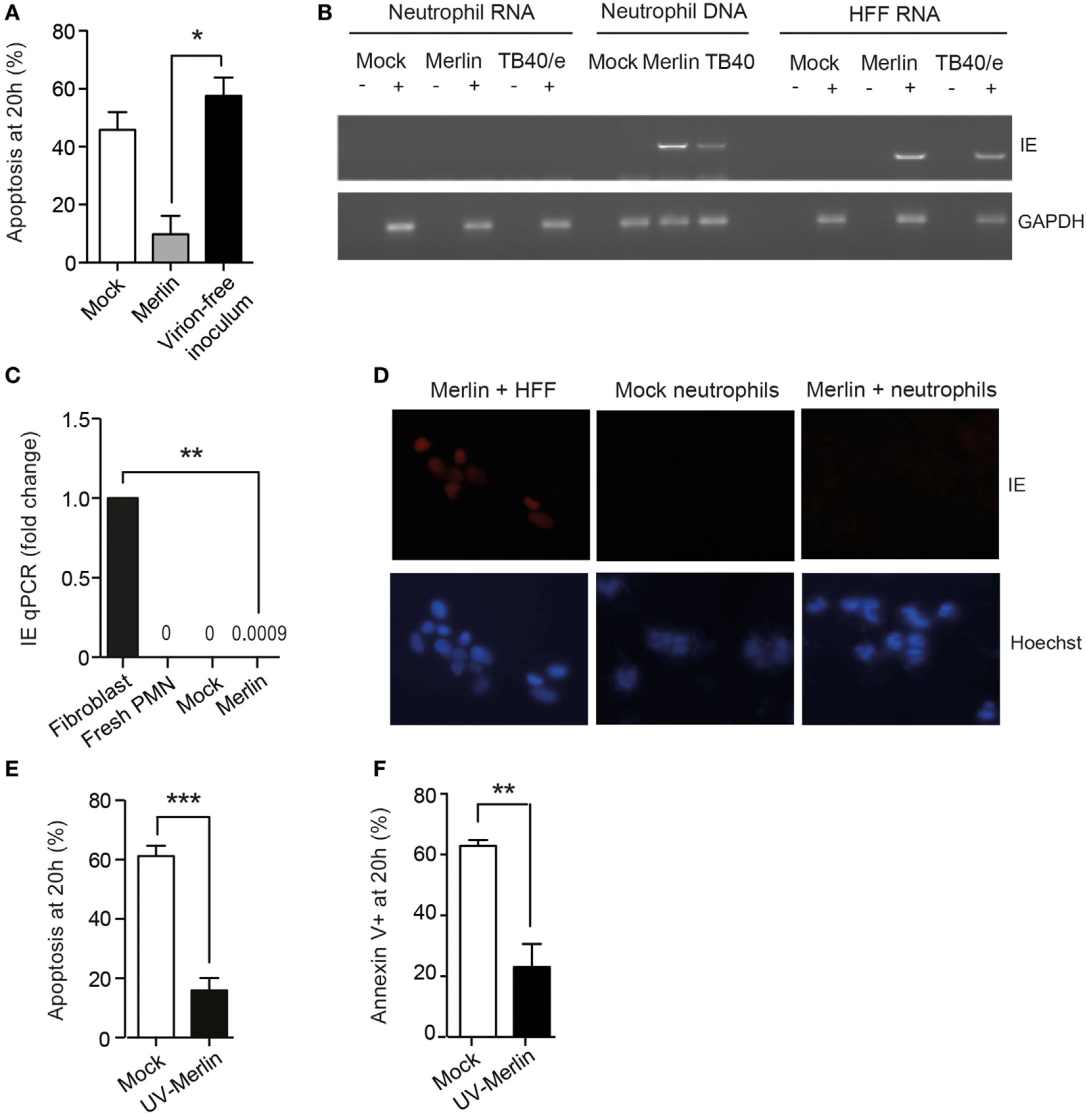
Human cytomegalovirus (HCMV) promotion of neutrophil survival is independent of viral gene expression. **(A)** HCMV Merlin inoculum was ultracentrifuged at 180,000 *g* for 2 h, and the supernatant was removed from the virion pellet and used to infect neutrophils (virion-free inoculum). Apoptosis was assessed at 20 h by morphology (*n* = 3) compared to mock-infected cells and cells infected with non-centrifuged Merlin inoculum (Merlin). **(B)** RNA or DNA was isolated from neutrophils and RNA from human foreskin fibroblasts (HFFs) 18 h after infection with HCMV strain Merlin, and RNA was subjected to reverse transcription, and all samples were then amplified in an IE72 PCR or GAPDH PCR control before loading onto the gel. **(C)** qPCR showing gene expression levels of IE72 RNA isolated at 6 h from neutrophils infected with Merlin versus control, relative to Merlin-infected fibroblasts (MOI 3, *n* = 3). **(D)** Neutrophils were mock infected or incubated with HCMV for 24 h and then stained for immediate early (IE) antigen, shown with Hoescht nuclear counter stain and Merlin-infected HFFs as a positive control. **(E)** Morphological (*n* = 6) and **(F)** flow cytometric analysis (*n* = 5) of neutrophil apoptosis in cells infected with Mock or UV-inactivated Merlin at 20 h (MOI 3). Data represent mean ± SE, **p < 0.01, ***p < 0.001.

Ultracentrifugation of the viral inoculum, to pellet and remove all infectious virus, removed the survival effect (Figure 2A), indicating that the virion itself, rather than substances present in the viral inoculum, was responsible for survival. Of note, HCMV-induced neutrophil survival was evident even in the absence of viral lytic gene expression. Hence, there was no evidence to support robust IE gene transcription within the neutrophil as judged by RT-PCR (Figure 2B) and only minute quantities detected by RT-qPCR (Figure 2C). Consistent with a lack of transcription, no IE protein was present on staining for IE expression 24 h postinfection (Figure 2D). Of note, neutrophil survival was equally evident with UV-inactivated virus (Figures 2E,F), implying that a virion component and not a viral protein synthesized *de novo* was responsible for the pro-survival effect. Thereafter, UV-inactivated HCMV was used for most subsequent experiments.

### HCMV-Induced Neutrophil Survival does not Require Internalization and Signals through ERK and NF-κB

**Figure 3:**
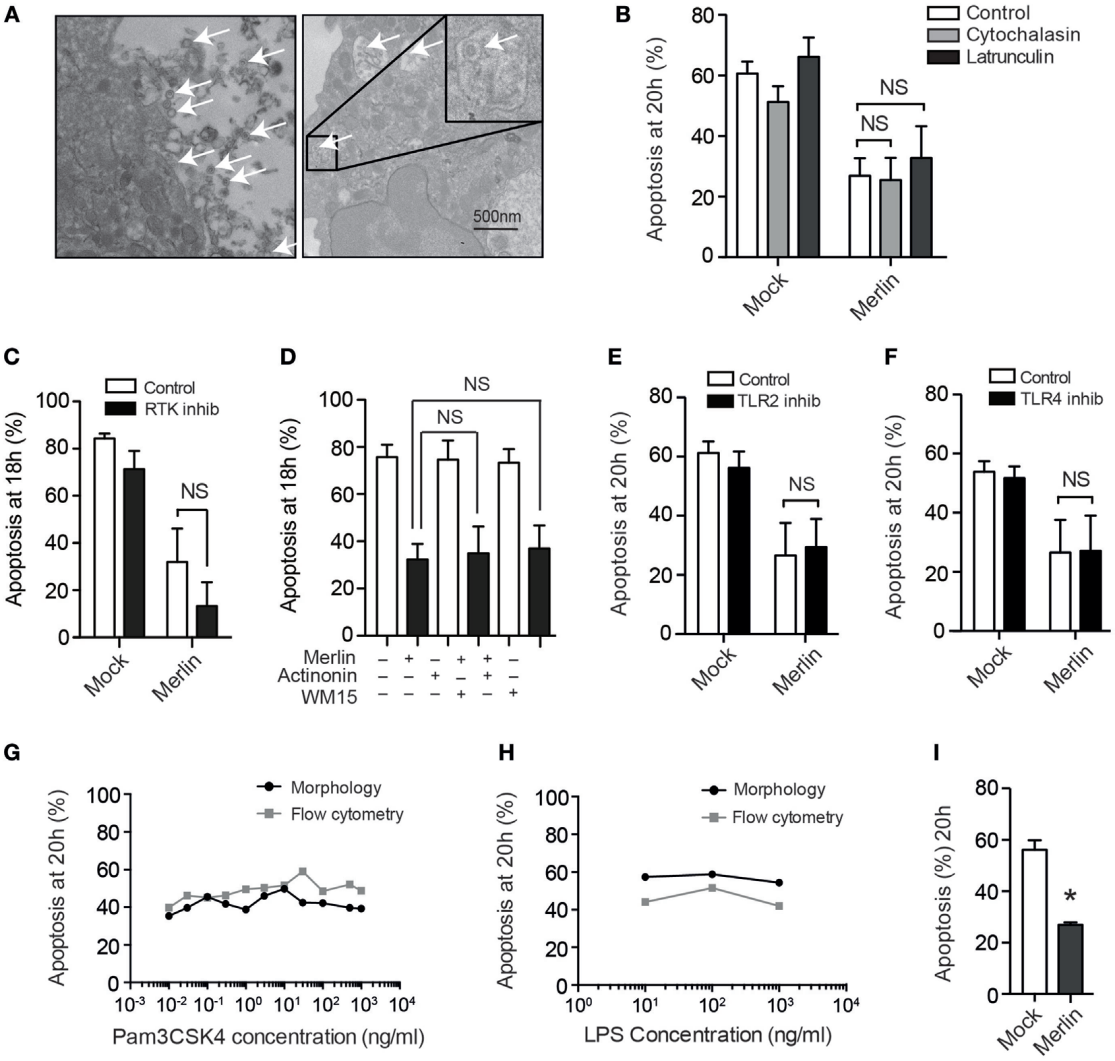
Human cytomegalovirus (HCMV)-stimulated neutrophil survival does not require internalization and does not signal through known receptors for HCMV. **(A)** Detection of viral particles (arrows) by transmission electron microscopy both outside and (inset) within HCMV strain Merlin-infected neutrophils 30 min postinfection (bar represents 500 nm). **(B)** Cytochalasin D or latrunculin A (10 μM) pretreatment did not influence neutrophil apoptosis following infection, compared with vehicle control (MOI 3, *n* = 3). **(C)** Morphological analysis of neutrophil apoptosis at 18 h in cells preincubated with the receptor tyrosine kinase inhibitor 324841 prior to Mock or Merlin infection (MOI 3, *n* = 3). **(D)** Morphological analysis of neutrophil apoptosis at 18 h in neutrophils pretreated with the CD13 inhibitors actinonin or anti-CD13 clone WM15 before Mock or Merlin infection (MOI 3, *n* = 3). Neutrophils were incubated with inhibitors of **(E)** TLR2 (*n* = 3) or **(F)** TLR4 (*n* = 3) prior to infection with Merlin (MOI 3), and apoptosis was assessed by morphology at 20 h. Dose–response curves are shown measuring neutrophil apoptosis at 20 h by cell morphology and flow cytometry using annexin V for **(G)** the TLR2 agonist Pam3CSK4 (*n* = 1) and **(H)** the TLR4 agonist LPS (*n* = 1). **(I)** Morphological analysis of neutrophil apoptosis in mock and Merlin-infected ultrapure neutrophils at 20 h (MOI 1, *n* = 3). Data represent mean ± SE, *p < 0.05. NS, not significant.

To investigate the mechanism by which neutrophil survival was mediated, we first set out to determine whether viral uptake was necessary to trigger survival signaling. While EM images taken 30 minutes postinfection revealed that very occasional HCMV virions are present within intracellular vesicles (Figure 3A), suggestive of phagocytic uptake, the majority of particles remained extracellular, and pretreatment of neutrophils with the phagocytosis/endocytosis inhibitors cytochalasin D or latrunculin A had no impact on the survival phenotype (Figure 3B). These observations suggested that HCMV-mediated neutrophil survival was dependent on a cell surface binding and/or postentry event mediated in the absence of viral gene expression. This was explored by inhibiting a number of receptors known to be activated by HCMV. Blockade of CD13 (Figure 3C) or EGF-R (Figure 3D), previously reported receptors for HCMV, did not reduce survival. The role of TLRs in neutrophil survival has been well documented (47), and HCMV has been shown to signal through TLR2 (48); however, no impact on survival was demonstrated with inhibition of TLR2 (Figure 3E). Furthermore, blockade of TLR4 signaling, another receptor known to be involved in viral detection and pro-survival signaling, again did not influence the pro-survival phenotype (Figure 3F). The inhibitors were shown to be effective in inhibition of TLR signaling by blocking IL-8 release by neutrophils in response to stimulation with agonists for TLR2 and TL4 (data not shown), but the agonists of TLR2 (Pam3CSK4) and TLR4 (LPS) themselves did not exert a pro-survival effect on neutrophils, further excluding the involvement of TLR2 and TLR4 in HCMV-induced survival (Figures 3G,H). We isolated ultrapure neutrophils (purity >99%) to mitigate any indirect effect of HCMV on contaminating monocytes, of which low numbers are present in our routine neutrophil preparations. The release of survival factors from contaminating monocytes has previously been noted to drive RSV-induced neutrophil survival (49, 50); however, our survival phenotype was still present in ultrapure neutrophil preparations (Figure 3I). The lack of any neutrophil survival effect when using mock transfected preparations, which had been prepared in an otherwise identical manner as the HCMV preparations, also excluded any inadvertent contaminant effect such as lipopolysaccharide.

**Figure 4:**
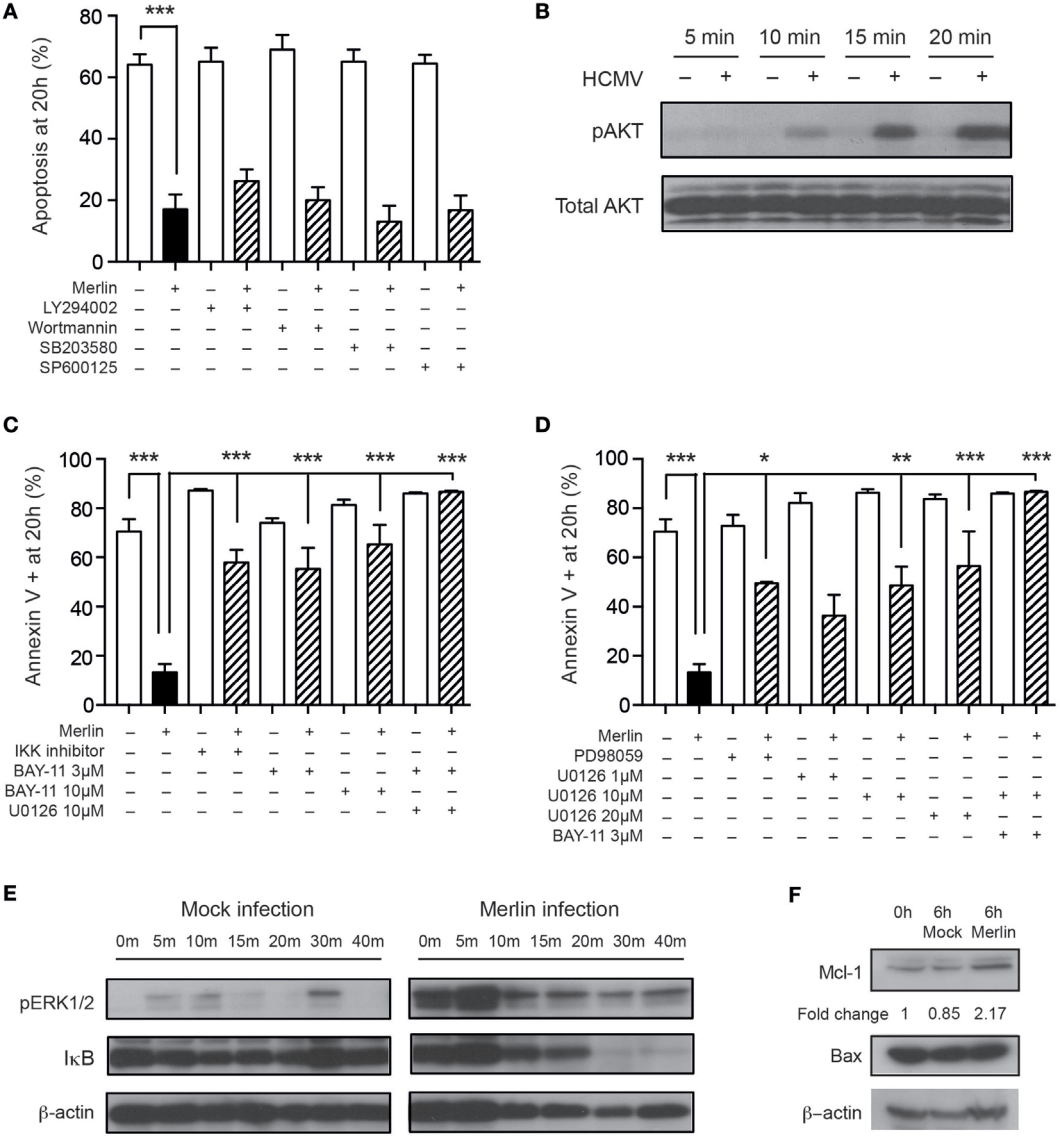
Human cytomegalovirus (HCMV)-induced neutrophil survival signals through multiple pathways. **(A)** Neutrophils were preincubated with the PI3K inhibitors LY294002 or wortmannin, the p38-MAPK inhibitor SB203580 or the JNK inhibitor SP600125 prior to infection with Merlin, and apoptosis was assessed by morphology at 20 h (MOI 3, *n* = 3–5). **(B)** Western blot analysis for total and phosphorylated AKT in neutrophils incubated with mock or HCMV at 5, 10, 15, and 20 min (MOI 3). **(C)** Neutrophils were preincubated with the NF-κB inhibitors IKK inhibitor VII or BAY-11-7085 (3 or 10 μM), with or without addition of the ERK1/2 inhibitor U0126, prior to HCMV infection, and apoptosis was assessed by flow cytometry using annexin V at 20 h (MOI 3, *n* = 2–4). **(D)** Neutrophils were preincubated with the ERK1/2 inhibitors PD98059 or U0126 (1, 10, or 20 μM), with or without the addition of BAY-11-7085, prior to infection with HCMV, and apoptosis was assessed by flow cytometry using annexin V at 20 h (MOI 3, *n* = 2–4). **(E)** Western blot analysis for ERK1/2 phosphorylation or IκB degradation was performed on mock or Merlin-infected neutrophils at 0, 5, 10, 15, 20, 30, and 40 min postinfection (MOI 3). **(F)** Western blot analysis for MCL-1 and Bax expression performed at 0 or 6 h after infection with mock or HCMV (MOI 3). Data represent mean ± SE, *p < 0.05, **p < 0.01, and ***p < 0.001.

Human cytomegalovirus activates a number of signaling pathways in both permissive and non-permissive cells, including the PI3K and ERK-MAPK pathways, identified previously as important for HCMV-mediated cell survival (51, 52), and the NF-κB pathway (53). We have previously published data indicating the importance of the PI3-kinase, MAPK, and NF-κB signaling pathways in neutrophil survival following GM-CSF and TNFα stimulation (44). Therefore, we used previously optimized concentrations of inhibitors of these pathways to assess involvement in the HCMV-induced neutrophil survival phenotype. Inhibition of the PI3K pathway, along with the p38-MAPK and JNK-MAPK pathways, did not affect HCMV-induced survival (Figure 4A), despite western blot showing activation of the PI3K pathway (Figure 4B). However, inhibition of both NF-κB (Figure 4C) and the ERK1/2 pathway (Figure 4D) led to a partial reduction in the HCMV survival effect, which was completely abrogated on blockade of both signaling pathways (Figures 4C,D). Consistent with these findings, Western blot analyses showed that HCMV treatment of neutrophils induced rapid ERK phosphorylation and IκB degradation (Figure 4E). ERK1/2 and NF-κB signaling have been implicated in the regulatory control of the balance between the pro-apoptotic protein Bax and the antiapoptotic protein MCL-1, the relative levels of which are crucial for the onset of constitutive neutrophil apoptosis (54), and Western blotting for these two proteins revealed stabilization of MCL-1 in response to HCMV infection but no change in Bax expression levels (Figure 4F).

### HCMV-Induced Neutrophil Survival is Viral Strain Specific

The pro-survival effect of HCMV on neutrophils was considered surprising, given the inability of HCMV to replicate efficiently inside the neutrophil. As such, the clinical relevance of this finding was unclear, i.e., whether this represents an antipathogenic mechanism employed by the neutrophil or a form of immune subversion by the virus. The fact that clinical strains of the virus retain the evolutionarily conserved chemokine vCXC-1, a potent IL-8 homolog and neutrophil chemoattractant, suggested that recruitment of this cell type may be beneficial to the virus in the host setting.

**Figure 5:**
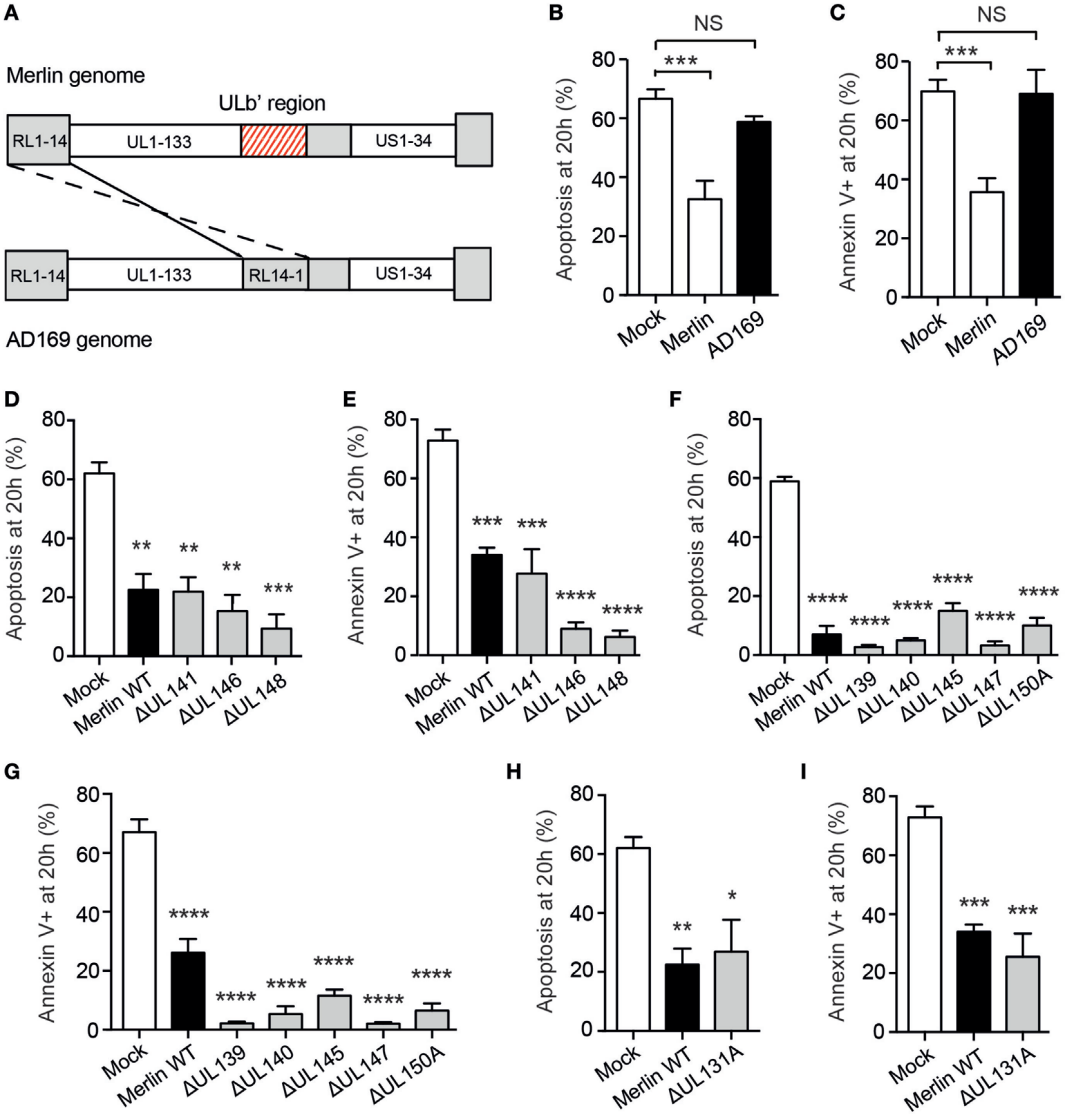
Human cytomegalovirus (HCMV)-induced neutrophil survival is viral strain specific. **(A)** The ULb′ region of the HCMV genome containing viral genes UL133–UL151 is lost in AD169 and replaced with inverted repeat RL14-1. **(B)** Neutrophils were incubated with the laboratory-adapted HCMV strain AD169 for 20 h, and apoptosis was assessed by cell morphology and **(C)** flow cytometry, compared to mock-infected neutrophils or neutrophils incubated with the clinical HCMV strain Merlin (MOI 3, *n* = 3–4). Neutrophils were incubated with wild-type Merlin or mutant Merlin strains lacking one of three genes from the ULb′ region of the HCMV genome known to code for virion components, and apoptosis was assessed at 20 h by **(D)** morphology and **(E)** flow cytometry (MOI 3, *n* = 3–4). Neutrophils were incubated with mutant strains lacking one of five ULb′ genes potentially coding for virion components, and apoptosis was assessed at 20 h by **(F)** morphology and **(G)** flow cytometry (MOI 3, *n* = 3). Neutrophils were incubated with wild-type Merlin versus Merlin lacking the UL131A gene, and apoptosis was assessed at 20 h by **(H)** morphology and **(I)** flow cytometry (MOI 3, *n* = 3). Data represent mean ± SE. **p < 0.01, ***p < 0.001, and ****p < 0.0001. NS, not significant.

To investigate this, neutrophils were co-incubated with the attenuated HCMV strain AD169, which has lost many of the genes carried in virulent isolates, including vCXC-1; this has been achieved through extensive passage resulting in the loss of multiple genes including the entire UL/b′ gene region encompassing UL133-UL151 (Figure 5A) (55). Rather strikingly, neutrophils incubated with AD169 showed no survival phenotype (Figures 5B,C), strongly suggesting that a viral locus within the ULb′ region or another gene mutated in this strain was responsible for the survival effect. Of these, nine genes are probable virion components (P Tomasec, personal correspondence), including UL146, a particularly strong candidate as it has been demonstrated to induce survival in human neutrophils (56), and UL141, which binds and downregulates the TRAIL death receptor on HFFs protecting them from extrinsic apoptosis (57).

To investigate the viral gene product responsible for HCMV-induced neutrophil survival, we screened a library of Merlin strains each containing a single-gene knockout covering the entire 19 gene ULb′ region. Surprisingly, all of these mutants induced neutrophil survival comparable to wild-type Merlin, including all nine genes thought to code for virion components (Figures 5D–G), indicating that the HCMV-induced neutrophil survival effect is not driven by a single ULb′ gene product. These data implicate either an additional gene mutated in AD169 outside the ULb′ region or a combinatorial effect of multiple gene products. One prominent candidate gene outside the ULb′ region is the UL131A locus, which is mutated in AD169 (58, 59), and in Merlin is involved in the formation of the viral pentameric complex required for HCMV entry into myeloid lineage and epithelial cells (60). However, when tested in isolation, the UL131A mutant also generated an intact survival response in neutrophils (Figures 5H,I), suggesting that the pentameric complex is not required for the survival phenotype.

### HCMV Infection Induces a Cellular Secretome that Further Potentiates Neutrophil Survival

**Figure 6:**
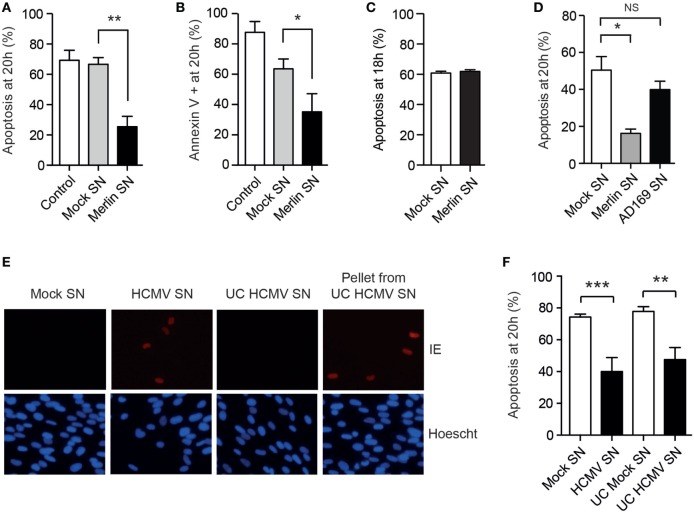
Human cytomegalovirus (HCMV)-exposed neutrophils produce a pro-survival secretome. Freshly isolated neutrophils were cultured with control media or supernatants from mock or Merlin-infected neutrophils (Merlin SN) collected at 20 h, and apoptosis was determined by **(A)** morphology and **(B)** flow cytometry at 20 h (MOI 3, *n* = 3). **(C)** Supernatant from mock or Merlin-infected monocytes was added to freshly isolated neutrophils and apoptosis determined by morphology at 18 h (MOI 3, *n* = 3). **(D)** Supernatant from mock or AD169-infected neutrophils was added to freshly isolated neutrophils and apoptosis determined by morphology at 18 h (MOI 3, *n* = 3). **(E)** Supernatant harvested from mock and HCMV-infected neutrophils at 20 h was centrifuged at 130,000 g for 2 h, and residual virus in the ultracentrifuged pellet and supernatant (UC HCMV SN) was assessed by transferring to fibroblasts for 24 h prior to staining for HCMV immediate early (IE) antigen, compared to mock SN and non-ultracentrifuged HCMV supernatant (HCMV SN) (representative image shown). **(F)** The ultracentrifuged supernatant was transferred to fresh neutrophils and survival assessed by morphology at 20 h compared to non-ultracentrifuged supernatant (*n* = 4–5, MOI 3). Data represent mean ± SE. *p < 0.05, **p < 0.01, ***p < 0.001.

Since events associated with HCMV binding and entry are by definition transient, we then considered whether a positive feedback loop might also operate to prolong neutrophil survival, hypothesizing that any secretome produced by the neutrophils in response to HCMV may propagate the survival phenotype. Consistent with this, virus-free supernatants derived from neutrophils exposed to the Merlin strain imparted a profound antiapoptotic effect when transferred to freshly isolated neutrophils (Figures 6A,B). Of note, supernatants prepared in an otherwise identical manner from HCMV-infected monocytes did not promote neutrophil survival (Figure 6C). Furthermore, supernatants from AD169-infected neutrophils did not convey a survival effect (Figure 6D), implying that this effect was restricted to clinical HCMV strains. The transferrable survival effect was not due to carryover of virus, as ultracentrifugation of supernatants to remove all virions (Figure 6E) caused no reduction in the survival conferred by the supernatant (Figure 6F), supporting instead a neutrophil-derived secreted factor.

**Figure 7:**
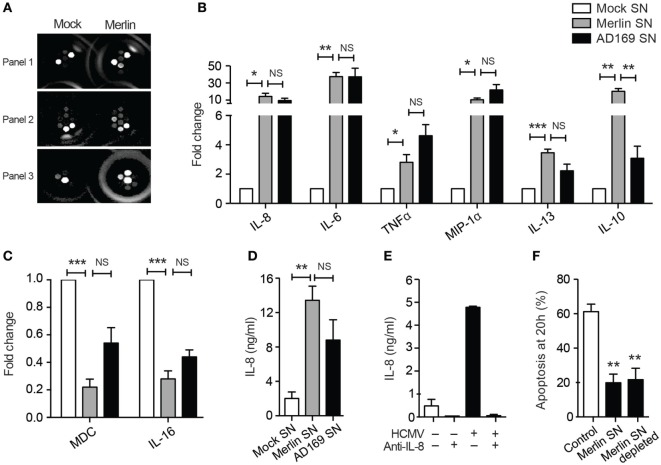
The human cytomegalovirus (HCMV)-exposed neutrophil secretome is high in IL-8. **(A)** MesoScale assay plate with three representative images, with each spot from three 10-plex wells representing one cytokine. The surrounding rings are imaging artifacts. **(B)** Results from MesoScale assay of mock and HCMV-infected (strain Merlin and AD169) neutrophil supernatants identified significant upregulation of IL-8, IL-6, TNFα, MIP-1α, IL-13, and IL-10 and downregulation of **(C)** MDC and IL-16 (MOI 3, *n* = 5). **(D)** IL-8 levels were measured in supernatants from mock- or HCMV-infected neutrophils at 20 h by ELISA (MOI 3, *n* = 5). **(E)** Following incubation with neutralizing antibodies against IL-8, supernatants were added to neutrophils and **(F)** apoptosis by morphology determined at 20 h, shown relative to control cells (MOI 3, *n* = 2–4). Data represent mean ± SE. *p < 0.05, **p < 0.01.

As the HCMV secretome appeared to elicit downstream effects that were acting in a paracrine manner, we consequently performed a MesoScale™ cytokine array on supernatants from mock and HCMV-treated neutrophils to try to identify potential pro-survival agents from a panel of cytokines (Figure 7A). Initial analysis revealed significant increases in the concentrations of the known pro-survival cytokines TNFα, IL-6, and IL-8 along with the chemokine MIP-1α and the immune-regulatory cytokines IL-13 and IL-10 (Figure 7B), together with decreased levels of the chemotactic molecules IL-16 and MDC (Figure 7C). There was no upregulation of other potential survival cytokines including GM-CSF, IL-1β, IL-2, IL-4, IFNγ, IL-12p70, and VEGF (data not shown), and the only cytokine significantly higher in Merlin-infected neutrophil supernatants compared to AD169 supernatants was IL-10 (Figure 7B). HCMV has been shown to stimulate IL-10 production in monocytes (61), but rather than being pro-survival IL-10 has been shown to induce apoptosis in neutrophils (62). Consistent with this, we were unable to detect any survival effect of recombinant human IL-10 on human neutrophils (data not shown).

We investigated IL-8, a recognized pro-survival cytokine, which was secreted in high concentrations in HCMV-exposed neutrophils as measured by MesoScale array and confirmed by ELISA (Figure 7D) and which showed a trend to increased expression in Merlin supernatants over AD169 supernatants. However, although IL-8 has been shown to cause a modest inhibition of neutrophil apoptosis (63, 64), we were unable to recapitulate this effect in our system using recombinant human IL-8 (data not shown), and crucially, antibody depletion of the endogenous IL-8 in the protective secretome using protein A beads (Figure 7E) had no effect on the transferrable survival (Figure 7F), indicating that IL-8 was not contributing to the survival effect observed.

### Interaction of HCMV with Neutrophils Promotes a Permissive and Immune-Suppressive Phenotype in Migrating Monocytes

Throughout these studies, the paradox between HCMV encoding a neutrophil chemoattractant and triggering marked neutrophil survival and heightened effector functions of neutrophils (32), and the minimal evidence that these cells represent a major site of productive infection or dissemination remains intriguing. While this could represent a host antiviral strategy, we speculated that the HCMV-induced neutrophil secretome may have a beneficial impact on the subsequent infection or activity of other cell types important for HCMV pathogenesis and dissemination. We set out to ascertain the effects of the neutrophil secretome on other immune cell types, predominantly the monocyte which plays a key role in HCMV dissemination and pathogenesis, to better understand the biological significance of HCMV prolonging neutrophil survival.

**Figure 8:**
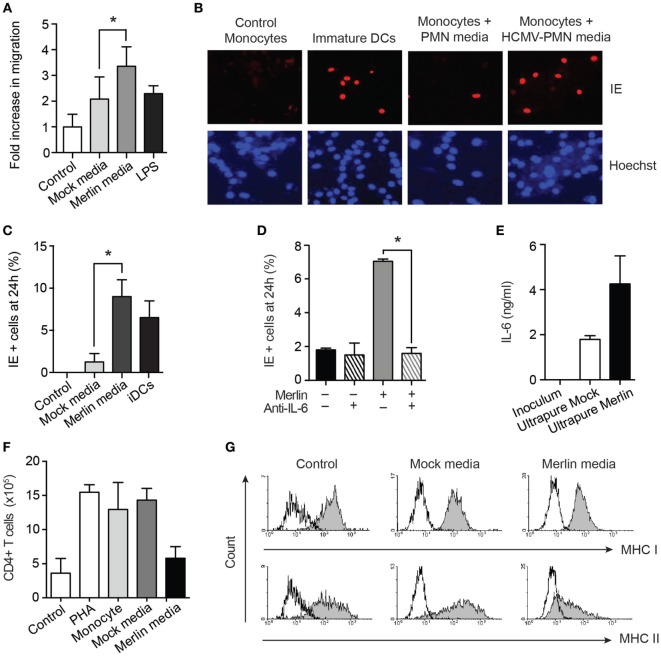
Supernatants from human cytomegalovirus (HCMV)-exposed neutrophils promote monocyte migration and activation to a permissive phenotype in an IL-6-dependent manner. **(A)** Monocytes were cultured in transwell plates in the presence of control media, or media from mock- or HCMV-infected neutrophils, or from LPS-activated monocytes as a positive control, and migration assessed by microscopy after 2 h (*n* = 3). **(B)** Autologous donor monocytes were cultured in control media, mock-infected neutrophil media, or Merlin-infected neutrophil media, and freshly infected with Merlin, alongside IL-4/GM-CSF-induced dendritic cells (DCs) as a positive control. Cells were stained for immediate early (IE) gene expression at 24 h and counterstained with Hoechst nuclear dye, and **(C)** relative levels of infection calculated from 10 fields of view (MOI 3, *n* = 3). **(D)** Neutralization of IL-6 in supernatant from mock or Merlin-infected neutrophils significantly inhibited the number of monocytes positive for IE gene expression (*n* = 3). **(E)** IL-6 levels by ELISA in viral inoculum and in mock-infected and Merlin-infected ultrapure neutrophils (purity >99%) (*n* = 2). **(F)** Monocytes were incubated with media (monocyte) or supernatants from mock- or Merlin-infected neutrophils for 3 days. Media was then removed, and allogeneic CD4+ T cells were added to each well along with phytohemagglutinin (PHA) to all conditions except control. T cell proliferation after 6 days of co-culture was assessed by counting and shown relative to T cells cultured alone (control) or in the presence of PHA alone (PHA) (*n* = 2). **(G)** Representative flow cytometry plots of autologous monocytes cultured in X-vivo-15 media (control), mock-infected neutrophil media, or HCMV-infected neutrophil media (MOI 3) for 3 days before staining for MHC class I and II. Data represent mean ± SE. *p < 0.05.

The elevated levels of the chemokine MIP-1α in the secretome (Figure 7B) suggested the potential for increase in monocyte migration. A transwell migration assay confirmed this to be the case (Figure 8A). The ability of neutrophils to enhance monocyte recruitment is a classical response to pathogen invasion; however, in this instance, migration of monocytes occurred together with transformation of the monocytes to a cell status permissive for HCMV infection, which is comparable to classical DCs generated *in vitro* (Figures 8B,C). This monocyte switch to a HCMV-permissive phenotype was dependent on the high levels of IL-6 in the secretome (Figure 7B), as demonstrated by a substantial reduction in this effect in the presence of a neutralizing IL-6 antibody (Figure 8D). IL-6 can be produced by monocytes and contaminating cells, so to eliminate the possibility that the IL-6 was a product of contaminating monocytes, or already present in the viral inoculum generated from HFF supernatants, an IL-6 ELISA was carried out on viral inoculum and on supernatants harvested from ultrapure neutrophil preparations infected with HCMV. IL-6 was not present in viral inoculum but was found in the ultrapure neutrophil supernatants, indicating that it is released from the neutrophils upon infection (Figure 8E).

The differential levels of IL-10 in our Merlin and AD169 supernatants, although not responsible for survival, also led us to investigate its effect on monocyte immune function. IL-10 has been shown to polarize monocytes to an anti-inflammatory M2 phenotype, with reduced MHC class II expression and decreased ability of monocytes to activate CD4+ T cells (65, 66). Consistent with this, our monocytes cultured in HCMV-exposed neutrophil supernatants failed to stimulate T cell division in a mixed leukocyte reaction (Figure 8F) and showed strong downregulation of MHC class II (Figure 8G). Thus, HCMV interaction with neutrophils was shown to promote the secretion of a cytokine profile that is chemotactic for monocytes and drives their subsequent activation to a HCMV-permissive and immune-suppressive phenotype.

## Discussion

The precise nature of the interaction of HCMV with neutrophils has been challenging to resolve. Although HCMV has been reported to heightened certain neutrophil effector functions (32) and HCMV can transfer to neutrophils following co-culture with permissive cells through microfusion events (31), the ability of neutrophils to support full lytic viral replication has never been shown conclusively, with viral cycling restricted to early replication events only (31, 32). The reason for this block in replication is not known. Although the detection of viral antigens has been identified in the neutrophils of viremic patients (25), this is likely to represent non-specific phagocytosis, because in healthy seropositive individuals, neutrophils do not appear to be a site of viral carriage (24). As such, the precise benefit of HCMV retaining vCXC-1 or why HCMV-infected permissive cell lines secrete the chemokines IL-8 and CXCL-1 and induce neutrophil chemotaxis (67, 68) is uncertain.

In this study, we sought to understand the nature of the interaction between HCMV and neutrophils at a cellular level, to elucidate the mechanisms involved in the neutrophil survival we observed, and to determine potential downstream effects of the virus–cell interaction on neighboring neutrophils and monocytes, to determine the potential significance of our findings *in vivo*. Herein, we show for the first time a pronounced effect of cell-free clinical HCMV isolates on the biology of this cell, an effect that is preserved in UV-treated virus but absent in an attenuated HCMV strain. We describe how the interaction of HCMV with neutrophils triggers a profound survival phenotype that involves ERK phosphorylation and IκB degradation and correlates with MCL-1 stabilization, along with the secretion of a bioactive secretome that promotes further neutrophil survival, monocyte chemotaxis, and development of monocyte permissiveness for HCMV infection.

Previous studies have reported that co-culture with HCMV-infected endothelial cells or fibroblasts protects neutrophils from apoptosis, at least in part due to IL-8 secreted by the co-cultured cells (32, 33). Skarman et al. described a co-culture system of neutrophils with HCMV-infected endothelial cells, in which the neutrophils showed reduced apoptosis (32); the authors concluded that neutrophil survival was linked to lytic gene expression due to the detection of IE antigen. In those experiments, neutrophils did not become positive for the main viral antigen pp65 when incubated with cell-free virus; hence, no investigation of neutrophil survival with cell-free virus was made. Furthermore, the study referred to endothelial cells infected with laboratory HCMV strains, which were then co-cultured with neutrophils; however, here, we report that direct inoculation of neutrophils with wild-type strains of HCMV promotes survival and demonstrate that the lab strain used in the previous study does not promote survival directly. A further study has also shown that, when co-cultured with HCMV-infected endothelial cells or fibroblasts, neutrophils display a reduction in apoptosis (33). Indeed, that study reported that the neutrophils also showed reduced apoptosis when co-cultured with non-infected endothelial cells and demonstrated that the survival was due to IL-8 produced by both infected and non-infected endothelial cells. Hence, the Skarman study failed to disentangle the effects of a secretome generated from a virally infected bystander cell from direct engagement of the neutrophil with cell-free HCMV.

The inhibition of neutrophil apoptosis may be significant in multiple ways for the host, as it has the potential to exacerbate tissue damage but also to aid viral clearance. Neutrophil influx to sites of viral infection is associated with inflammation and tissue damage in influenza (69) and RSV (70). It is suggested that increased neutrophil viability may lead to greater HCMV-induced tissue damage in PCR-positive inflammatory bowel disease (32, 71), and the observation that neutrophil survival is absent with the attenuated HCMV strain AD169 would support the hypothesis that it is a virulence mechanism.

Conversely, in murine CMV infection, neutrophils form a critical part of the antiviral response and are shown to play a direct role in controlling viral replication and limiting morbidity (17), a role also seen in RSV (72), HSV (15), and HIV infection (73). Interestingly, neutrophils support viral replication in West Nile virus infection and are actively recruited during infection, acting as a reservoir for replication, and only become involved in viral clearance at a later stage (74). The presence of vCXC-1 and other chemokines would support a similar role in HCMV.

The potential benefit to the virus of a reduction in neutrophil apoptosis may be multifaceted. Prolonged neutrophil survival can delay the adaptive immune response, through a reduction in formation of apoptotic vesicles that would otherwise be ingested by monocytes or DCs and trafficked to lymph nodes for antigen presentation, as occurs in *Mycobacterium tuberculosis* (12). In addition, despite limited viral replication in neutrophils, there is evidence that the neutrophil may be used by the virus for dissemination. Circulating HCMV-infected neutrophils may act as a “Trojan horse” to infect other cell types, as neutrophils infected by co-culture are seen to transmit to naive permissive cells *via* cell-to-cell transfer of viral particles, even up to 48 h after initial separation (33). HCMV infection often results in a concerted antiapoptotic response to prolong host cell lifespan for replication, and during lytic infection, the virus encodes an array of antiapoptotic proteins that target caspase-8 (UL36) (75), mitochondrial membrane stability (UL37 × 1) (76), ER stress (UL38) (77), ATP production (b2.7) (78), and p53 (IE2) (79). The survival effect we observe in neutrophils, involving a likely virion–cell surface interaction, is similar to that observed during non-permissive infection of CD14+ and CD34+ cells (51, 52), where ERK and PI3K signaling were shown to be key. In that study, HCMV glycoprotein B was an important agonist of the survival response in CD34+ cells, yet this seems unlikely to be the case here since AD169 is not protective. It is possible that the model proposed for the survival of monocytes, which requires multiple signaling events, could be relevant in the neutrophil (80).

Furthermore, the cytokine profile we see here released by the longer-lived HCMV-exposed neutrophils appears to provide a further advantage to the virus through further potentiation of neutrophil survival and through effects on circulating monocytes, through recruitment, differentiation, and activation to render these cells permissive for HCMV. We were unable to identify the survival factor(s) present in the secretome, as our screen of 29 cytokines did not highlight any prosurvival factors upregulated in Merlin supernatants and an array of 39 more growth factors and soluble receptors failed to identify any other significantly upregulated factors (data not shown). However, we were able to pinpoint IL-6 as a contributor to monocyte permissivity. The reactivation of HCMV has been previously reported from monocytes activated by cytokine stimulation including IL-6 (81), suggesting that limited monocyte differentiation/activation may be sufficient to trigger a permissive phenotype, which is consistent with our data. However, acute treatment with recombinant IL-6 has not been shown to induce monocyte permissiveness for HCMV infection alone (82), implying that either the effects of IL-6 are chronic long-term effects or, in fact, may be working in concert with other factors. A likely candidate is IL-13, which we observed the upregulation of in our secretome analyses. IL-13 is a member of the IL-4 cytokine family and has been shown to support monocyte differentiation (83)—a hypothesis consistent with the knowledge that differentiation is a key determinant of myeloid cell permissiveness for HCMV infection (84).

In the context of a HCMV infection *in vivo*, the induction of a pro-survival secretome is highly suggestive of a host antiviral response, so this appears to represent an antiviral strategy of unexpected benefit to HCMV. Neutrophils are among the first cells to traffic to the site of primary HCMV infection, with cross-cellular communications a crucial component of the initial response. This in turn leads to monocyte recruitment, to promote clearance of apoptotic debris generated by the initial neutrophil response, which here would facilitate enhanced dissemination of HCMV, a process in which monocytes are argued to be key (85, 86).

The absence of the phenotype with the non-virulent strain AD169 implies that the survival effect is due to a virulence gene, non-essential for growth, which has been lost or mutated in AD169 through extensive passage. Unfortunately, our screen of ULb′ knockouts did not identify any single gene to be responsible, and AD169 carries many other mutations besides the ULb′ deletion that could be responsible. We included a Merlin mutant lacking UL131A, which lies just outside the ULb′ region and is essential for the formation of the intact gH-gL-UL128-UL130-UL131A pentameric complex, required for leukocyte tropism and entry; although UL131A carries a truncation mutation in AD169 and would seem a likely candidate, once again no change to the phenotype was seen. Repeated attempts to generate a knockout of the whole ULb′ region against a Merlin backbone have not been successful to date, but if available in the future, such a mutant would help to address the question of whether the survival effect is due to a combinatorial effect of ULb′ genes, or another gene mutated in AD169 and present in the virion. HCMV contains at least 59 structural proteins (87), including the antiapoptotic UL38, and some of these carry mutations in AD169, including the membrane glycoprotein O (UL74) that forms the gH-gL-gO entry complex (88, 89). Of particular interest are UL36, an inhibitor of caspase-induced apoptosis (75), its antiapoptotic intronic micro-RNA mir-UL36-5p (90), and the pro-survival non-coding RNA b2.7 (78). All three are present in or carried on the virion (91, 92), carry at least minor mutations in AD169 (88, 89, 91), and are targets for further investigation.

In summary, we have demonstrated for the first time that the HCMV virion has a profound effect on neutrophil biology, delaying apoptosis and inducing the release of a highly bioactive secretome with the capacity to propagate neutrophil survival and to induce monocyte chemotaxis and differentiation to a permissive, anti-inflammatory phenotype. In the context of clinical infection, this is likely to represent a host response that provides an unexpected benefit to the virus, and although significant resolution of infection is likely to occur, differentiating monocytes provide the ideal vehicle for viral dissemination and the establishment of a lifelong persistent infection. A persistent infection without pathology in the host would, in evolutionary terms, represent an ideal scenario for the virus. This report highlights how the interaction of a viral pathogen with a cell type not capable of supporting lytic infection can still have a pronounced effect on both the biology of that cell and the wider immune response.

## Ethics Statement

This study was carried out in accordance with the recommendations of the Cambridge Local Research Ethics Committee (06/Q0108/281) with written informed consent from all subjects. All subjects gave written informed consent in accordance with the Declaration of Helsinki. The protocol was approved by the Cambridge Local Research Ethics Committee.

## Author Contributions

JP, DS, JJ, MR, EC, and AC designed and performed experiments and analyzed data. JP wrote the manuscript with input from MR. MR, MW, AC, and EC all provided editorial input. All authors contributed to the drafting and revising of the manuscript and have approved the final version.

## Conflict of Interest Statement

The authors declare that the research was conducted in the absence of any commercial or financial relationships that could be construed as a potential conflict of interest.
